# Use of Telemedicine Healthcare Systems in Pediatric Assistance at Territorial Level: Consensus Document of the Italian Society of Telemedicine (SIT), of the Italian Society of Preventive and Social Pediatrics (SIPPS), of the Italian Society of Pediatric Primary Care (SICuPP), of the Italian Federation of Pediatric Doctors (FIMP) and of the Syndicate of Family Pediatrician Doctors (SIMPeF)

**DOI:** 10.3390/jpm13020198

**Published:** 2023-01-22

**Authors:** Susanna Esposito, Cristiano Rosafio, Francesco Antodaro, Alberto Argentiero, Marta Bassi, Paolo Becherucci, Fabio Bonsanto, Andrea Cagliero, Giulia Cannata, Fabio Capello, Fabio Cardinale, Tiziana Chiriaco, Alessandro Consolaro, Angelica Dessì, Giuseppe Di Mauro, Valentina Fainardi, Vassilios Fanos, Alfredo Guarino, Giada Li Calzi, Elisa Lodi, Mohamad Maghnie, Luca Manfredini, Emanuela Malorgio, Nicola Minuto, Maria Grazia Modena, Rossano Montori, Andrea Moscatelli, Elisa Patrone, Elena Pescio, Marco Poeta, Angelo Ravelli, Maddalena Spelta, Agnese Suppiej, Sergio Vai, Luca Villa, Rinaldo Zanini, Renato Botti, Antonio Vittorino Gaddi

**Affiliations:** 1 Pediatric Clinic, Department of Medicine and Surgery, University of Parma, 43126 Parma, Italy; 2 Family Pediatrics, AUSL Modena, 41124 Modena, Italy; 3 Department of Pediatrics, IRCCS Giannina Gaslini, 16147 Genoa, Italy; 4 Family Pediatrics, 50139 Florence, Italy; 5 Alma Mater University, 40126 Bologna, Italy; 6 Family Pediatrics, 10121 Turin, Italy; 7 UO Territorial Pediatrics, Primary Care Department, AUSL Bologna, 40126 Bologna, Italy; 8 UOC of Pediatrics and ED with an Allergy-Pneumological and Immuno-Rheumatological Focus, Giovanni XXIII Pediatric Hospital, University of Bari, 70124 Bari, Italy; 9 Health Department, ASL Roma 3, 00125 Rome, Italy; 10 General Management, IRCCS Istituto Giannina Gaslini, 16147 Genoa, Italy; 11 Pediatric and Rheumatology Clinic, IRCCS Istituto Giannina Gaslini, 16147 Genoa, Italy; 12 Department of Neurosciences, Rehabilitation, Ophthalmology, Genetics and Maternal-Child Sciences (DINOGMI), University of Genoa, 16126 Genoa, Italy; 13 Department of Surgical Sciences, University of Cagliari, 09127 Cagliari, Italy; 14 Family Pediatrics, 81100 Caserta, Italy; 15 Section of Pediatrics, Department of Translational Medical Sciences, University of Naples Federico II, 80131 Naples, Italy; 16 P.A.S.C.I.A. Center (Heart Failure Care Program, Childhood Heart Diseases and Those at Risk), University of Modena and Reggio Emilia, AOU Polyclinic of Modena, 41124 Modena, Italy; 17 Pediatric Pain and Palliative Care Service, IRCCS Istituto Giannina Gaslini, 16147 Genoa, Italy; 18 UOC Anesthesia and Intensive Care, IRCCS Istituto Giannina Gaslini, 16147 Genoa, Italy; 19 UOSID Trial Center, IRCCS Giannina Gaslini Institute, 16147 Genoa, Italy; 20 Scientific Directorate, IRCCS Istituto Giannina Gaslini, 16147 Genoa, Italy; 21 Pediatric Clinic, University of Ferrara, 44124 Ferrara, Italy; 22 Center for Metabolic Diseases and Atherosclerosis, University of Bologna, 40126 Bologna, Italy

**Keywords:** telemedicine, teleconsultation, telepediatrics, telemonitoring, televisit

## Abstract

Technological innovation can contribute to a reorganization of healthcare, particularly by supporting the shift in the focus of care from the hospital to the territory, through innovative citizen-centered models, and facilitating access to services in the territory. Health and social care delivery modalities, enabled by telemedicine, are crucial in this regard. The objective of this Consensus document, written by the main Italian Scientific Societies involved in the use of telemedicine in pediatrics, is to define a standard for its use at the territorial level in various declinations in the pediatric field; this paper also identifies priority areas for its application and the types of services that most require intervention and investment. The changes that are underway in digital transformation in all sectors are unstoppable, and for the digital transformation to take place in a productive sense, the contribution of not only all health professionals, but also of patients, is necessary. From this perspective, authors from different backgrounds were involved in the drafting of this Consensus and, in the future, other figures, primarily patients, are expected to be involved. In fact, this belongs to the vision of connected care, in which the citizen/patient actively participates in the treatment path so that they are assisted in a personalized, predictive and preventive way. The future scenario must be able to provide for the involvement of patients from the initial stages of planning any treatment path, even in the pediatric age, and increasing, where possible, the proximity of the health service to the families.

## 1. Introduction

The change in the health needs of the population requires a structural and organizational redesign of the network of services, especially with a view to strengthen the territorial scope of assistance [1]. Technological innovation can contribute to a reorganization of health care, in particular, by supporting the shift in the focus of health care from the hospital to the community, through innovative care models centered on the citizen and facilitating access to services in the community [2,3,4]. The delivery methods of health and social-health services, enabled by telemedicine, are fundamental in this sense; they help to ensure a fair access to treatment in remote areas, support for the management of chronic conditions, a channel for accessing a highly specialized continuity of care through multidisciplinary comparison and a fundamental aid for emergency services [5,6].

There are many telemedicine initiatives at a national and international level that, too often, however, can be traced back to experiments, prototypes and projects that are characterized by limited cases and high mortality rates. In the face of this non-organic diffusion of health services provided by telemedicine methods, it is necessary to have a shared governance model of telemedicine initiatives; this must be the central point in the specific knowledge of the health sector. These limitations, together with the need to include indicators capable of describing the performance of the different initiatives, represent gaps in telemedicine implementation for the improvement of the health service. A harmonization of the guidelines and application models of telemedicine is, therefore, necessary; this is as a prerequisite for the interoperability of telemedicine services, and is a requirement in the transition from the experimental logic of using telemedicine services to the structured logic and widespread use of these services. This Consensus document is a narrative review written by the main Italian Scientific Societies involved in the use of telemedicine in pediatrics. Its objective is to define a standard for telemedicine’s use at a territorial level in various declinations in the pediatric field, and identify priority areas of application, types of services, services with the greatest need for intervention and investment, and performance indicators and future directions.

## 2. Telemedicine at Home

### 2.1. The Patient’s Home as a Place of Care

When, for various reasons (e.g., disability, isolation due to contagious disease, significant distance between home and outpatient clinics, exceptional climatic imperviousness), a proximity between the patient and the primary care pediatrician is extremely difficult, if not impossible, the patient’s home must become the first place of prevention and care, where the person can more easily become aware of his or her own health status; this is also through health and social care, and can, therefore, manage the care he or she receives with greater awareness [7]. In these situations, the home represents not only the place where the patient is well, but also the opportunity for great financial savings. It is a place where the concept of proximity and personalization in the care program becomes a reality, especially when it comes to chronic and disabling diseases. In particular, it offers the possibility of measuring all the interventions within the care plan by bringing about the work activities of the different local stakeholders that were previously unmeasurable [8].

With this perspective, there is a need to develop clinical, organizational and management governance tools, such as the development of digital systems and platforms capable of sharing information about the person being cared for in real time; this includes the adoption of uniform multidimensional assessment systems that are capable of measuring the state of frailty, elaborating prognostic indications and estimating the intensity of care that Individual Care Plans will have to ensure for the patient [9].

For this goal to be achieved, high quality connectivity is needed. There is a need to design shared criteria for analyzing home spaces to assess their potential and suitability, in relation to the individualized care plan. There is a need for a training pathway to ensure that health care personnel and families are adequately trained. Finally, it is necessary to have an integrated and interconnected pathway to connect healthcare facilities, community resources, and care tools (including medical devices) and, on the other hand, to develop the use of intercommunicable and expandable modular software and platforms [10]. The scalability of these resources must be a fulcrum, around which the journey of health data is planned and that must become circular and valuable information. Finally, it is necessary to define the empowerment programs aimed at patients, in terms of skills, digital and otherwise, without excluding patients’ family members and/or caregivers in general. Such programs must not only be contributed to by health professionals in their drafting, but also by the representatives of the different categories of patients, especially in relation to chronic pathological conditions with a multi-year course [11,12].

### 2.2. The Virtual Hospital

The development of health knowledge, and the possibility of accessing increasingly advanced and sector-specific preventive, diagnostic-therapeutic, rehabilitative and care procedures, presents the need to develop more accessible networks of intervention and access to the health care system. Currently, specialist care and access to second- and third-level diagnostic-therapeutic resources is mainly polarized in hospitals, with the need for families and minors to physically access (according to different modalities, such as emergency admission, scheduled hospitalization, day-hospital, specialist outpatient clinic) hospital facilities where specialists and the necessary instrumentation for the case management are located [13].

The development of a hospital-centric system may pose some advantages, but it often creates a gap with community medicine and the everyday life of the child, which cannot always be bridged by traditional health services. The very management of the network (family, school, health, educational and recreational services), and the sharing of information and actions among the various actors involved in it, is often cumbersome, unmanageable and unoptimized. The use of a telepediatrics model may be able to overcome some of the obstacles present in our health care system for health access for the pediatric population, through communication and data sharing systems that can take advantage of information and communication technologies. These goals can be achieved through the use of dedicated instrumentation, or by leveraging and integrating the system with technology that would normally be available in the population (personal computers, phones, smart-devices); these must be compatible with the limitations and precautions outlined below. The possible intervention scenarios of a network capable of exploiting the potential of information and communication technologies can be numerous [13]:

- The formation of a multidisciplinary network for the management and promotion of child health that encompasses all professional figures, including educational and nonhealthcare figures who revolve around the child and collaborate in his or her proper growth and development. In this regard, the communication network can enable the exchange of information in real time and in an optimized manner, with different levels of access and confidential privileges that vary according to the figure involved. The purposes are mainly health promotion, disease prevention and the early recognition of risk situations in social and health areas. The figures involved are the child/youth, the family, educational figures, and professionals in the world of childhood. The latter include the following: health figures (free-choice pediatrics, medical specialists, family nurses, health workers, psychologists, physiotherapists, health technicians); educational figures (school principals, teachers and educators, non-teaching school personnel); continuing educational services (trainers, educators, teachers, coaches); social services; and child support and assistance organizations and associations.
- The formation of a multidisciplinary network for chronicity management and health care that can share real-time information, actions, interventions and critical issues in the management of the child/youth with chronic or recovering conditions. The figures involved overlap with those described above. Levels of intervention and information exchange follow different streams, as shared data may be essential for individual case management (as in monitoring vital parameters) and actions taken may be dictated by specific needs that require different levels and priorities for intervention.
- Communication between health professionals with the possibility of managing exchanges and information flows in real time or not (synchronous or asynchronous communications) that allow access, even remotely, to diagnostic, therapeutic, rehabilitative and care resources normally available only in second- and third-level hospital facilities. The flow of information can move from primary care to ultra-specialization and vice versa; this transfers in the first case, the potential of the network of free-choice pediatricians and territorial specialists to the hospital, and, in the second case, the resources of the hospital hubs to the territory, where the diagnostic–therapeutic–assistance prescriptions are put in place.

The use of information and communication technologies in the pediatric field can help overcome physical and geographic limitations, going beyond the need for physical access to health care facilities in order to take advantage of their potential. However, the creation of a virtual environment, in which hospitalization in a facility or in-person access to specialized outpatient clinics is no longer necessary, creates critical issues that need to be taken into account, considering the peculiarities and vulnerabilities of the pediatric population to which these services are dedicated. The basic prerequisites for the creation of a Virtual Hospital that can take advantage of the network of the territory, including not only the health aspects, but all the services to children mentioned above, can be summarized schematically in the following points [7]:

- informed consent, which must be offered by the child’s parent/guardian and shared with the child, consistent with their age and ability to understand;
- the protection of the privacy of those involved, including in the future; this is because the data collected are initially managed by a guardian who takes charge of the child, but will become the property of the person concerned when he or she comes of age;
- the protection of the child’s dignity and rights;
- cybersecurity and the protection of data exchange (anamnestic information, vital parameters, reports, prescriptions, recommendations, letters, minutes, text documents, legal documentation, images, video footage, instrumental diagnosis, and data analysis, including for research purposes where the identity of the child may be revealed); access to this should be granted only to those with the appropriate credentials, according to the levels of confidence and privileges that vary according to the professional figure involved, and the purpose of intervention, with the possibility of bypassing the access block based on specific emergency situations and known needs [14];
- the use of reliable and accessible instrumentation that can ensure equal and nondiscriminatory access to care;
- the use of age-compatible software and hardware that is optimized and calibrated to the needs, understanding and natural inclinations of the child in question;
- integration with traditional care services, in an optimized manner in order to reduce the “burden” related to health care and specific diseases and health conditions; this is also to optimize the achievement of measurable goals regarding prevention and health promotion;
- the reduction in redundant cycles and the computerization of data collection, with the overcoming of the model and paper forms for the collections, management, sharing, distribution and processing of the data itself, and for the optimization of communications between the professionals involved;
- the use of flexible telepediatrics models that can adapt to the evolution of information and communication systems, and to the needs of the individual user, the health system, and society;
- the creation of feedback systems that can automatically or semi-automatically collect data on outcome indicators, with the aim of readjusting the model used to improve performance and overcome any critical issues.

### 2.3. When in-Patient Visit and Hospitalization Are Indicated

The implementation of telemedicine for inpatient care requires the standardization of clinical criteria that guides care pathways among the parties involved (children, families, pediatricians of free choice, hospitals, nurses, and specialists at referral centers). The following are some directions relevant to clinical practice [8]:

- priority areas of application -> indications for in-patient visitation and indications for admission apply to first-, second-, and third-level pediatrics. These indications are generically determined by clinical parameters (severity and urgency), the age and comorbidities of patients, and must necessarily take into account local health resources, pathways, their accessibility and risk of infection;
- the organizational model -> the organizational model must be shared among the different figures involved (families, pediatricians, hospitals, operating centers, third level centers). Recently, the Italian Federation of Pediatric Physicians (FIMP) proposed a policy document for the development of telemedicine in the care setting of Family Pediatrics, which can be a starting point for those approaching the use of such tools. It seems appropriate, in fact, to develop indications for the in-patient visit and hospitalization using organizational models that need to be implemented with continuous audit systems (Plan-Do-Study-Act type, PDSA);
- information and training aspects -> constitute a crucial element for the optimization of telepediatrics and its proper implementation. A cascade training of health professionals (primarily nurses) should be assumed. In this regard, the involvement of the Italian Society of Medical Pedagogy (SIPEM) seems useful. It is, in addition, appropriate to consider some institutional venues for pilot experiences (e.g., universities) where innovative and educational aspects are “institutionally” combined. A relevant aspect is the involvement of families in training (possibly in the context of health budgets). Caregivers need to be trained and confident in the use of such tools that enable the caregiver to be able to quickly identify clinical parameters and/or red flags that indicate the urgency of an in-person visit and/or hospitalization;
- performance indicators -> are specifically part of the PDSA methodology and should be determined at the local level (region, local health authorities, district). They should be distinguished into primary and secondary, and divided into health and organizational/economic indicators. It is essential that they be defined in the timeline and evaluated periodically in order to identify the possible barriers and interventions in order to overcome them. Such indicators include the number of admissions and number of visits, drug prescriptions, response times, and satisfaction questionnaires, among others;
- critical issues -> regulatory aspects, and the legal and insurance implications of telemedicine, are still poorly defined and could be framed in pilot or experimental initiatives.

Taking the above together, Table 1 shows the indicators for an in-person visit and Table 2 shows the indicators for hospitalization.

### 2.4. Psychological Support and Empowerment Support

The accelerating pace of life in the digital age and, in recent years, the changes in lifestyle and social relationships imposed by the pandemic, are increasing the risk of psychological consequences in pediatric and adolescent ages. The need for psychological support is increasingly recognized and sought after, and telemedicine may provide an important opportunity to improve access to care, cost, and quality of care. Tozzi and colleagues, in a survey given to families of children with Down syndrome, Williams syndrome, and 22q11 deletion, documented a positive attitude toward the use of telemedicine to support mental health [15].

In pediatric and adolescent ages, psychological support can be provided in a mixed modality, with initial in-person knowledge meetings and a follow-up in telemedicine, in the context of a televised setting. This allows for more accessible and direct communication with the caregiver. Consequently, the child and parents can feel more supported, assisted and favored in becoming protagonists in the management and monitoring of their child’s psychological well-being. Telemedicine, in fact, promotes what is known as empowerment, that is, the possibility that the parent, but also the older child and adolescent, become firsthand participants in the care process and acquire a greater awareness of their own state of psychological well-being and the required actions to improve it. A recent randomized controlled trial, conducted in children with attention-deficit/hyperactivity disorder (ADHD), compared the treatment delivered in telemedicine with hybrid and in-presence approaches; this demonstrated a better outcome in children treated with telemedicine [16]. In a subsequent study, the same group reported lower stress indices and better empowerment scales in the caregivers of children treated by telemedicine, compared with the traditional in-presence approach [17]. Remote psychological support also has the advantage of reducing geographic disparities by allowing mental health to be promoted according to the best standards of care in hard-to-reach areas [18]. In addition, in the context of psychological distress, so-called tele-assistance plays an important role and is especially useful in situations where distress and social isolation are associated [18]. In tele-assistance, psychologists and social workers can provide remote support to families, not making them feel “abandoned”, but supported and safe.

### 2.5. Physical Therapy, Rehabilitation, and Home Care

The priorities of pediatric rehabilitation and how these can be met with digital solutions start with the assumption of the main institutional documents involved in telemedicine [7] and telerehabilitation [14]. In addition, they agree on the centrality of multidimensional assessment, based on the International Classification of Functioning, Disability and Health (ICF), from which to prepare an individual rehabilitation project (PRI) to frame the telemedicine services needed and usable by the patient according to his or her characteristics.

The main causes of chronic or temporary disability affecting the pediatric population with motor rehabilitation needs include the following: orthopedic diseases of a congenital, post-traumatic, and neoplastic nature, as well as neurological disorders, such as infantile cerebral palsy (ICP), severe acquired cerebral palsy (GCA), spinal cord injury, and brain neoplasia [19,20].

The main evidence on rehabilitation services that can be delivered by telemedicine concerns:

- the televisit -> which can increase access to specialist visits at a low cost and from remote locations, ensuring high levels of caregiver satisfaction and the remote reproducibility of the assessment of some of the key clinical parameters [21,22,23,24];
- synchronous and asynchronous telecounseling -> improves the usability of services and caregiver satisfaction, with evidence of non-inferiority to the traditional treatment of infantile cerebral palsy and GCA [25,26,27,28,29,30,31] and showing potential efficacy. However, there is need for further clinical studies;
- telecounseling -> has been shown to be effective on the behavior of children with disabilities, particularly by intervening with parents with respect to the performance of the Home Exercise Program (HEP) [23,31];
- telemonitoring -> device-based and/or video-call-based; it can potentially provide feedback to healthcare professionals and patients, in order to modify the individual rehabilitation plan, and increase engagement and adherence to HEP [32,33].

The greatest certainty comes from the potential of improving HEP adherence, central to chronicity, and the remote delivery of counseling and follow-up visits, with positive impacts on parent-caregivers. Future prospects point to the use of digital therapies, particularly based on artificial intelligence, virtual reality/exergames, and wearables in the home with wearable settings [34,35,36]. There is an urgent need to define effective, reproducible, yet customizable digital rehabilitation protocols, to consolidate the supporting role of telerehabilitation alongside traditional methods, and to explore the prospects of substituting these methods in specific patient populations (Table 3).

### 2.6. Telemedicine and School: The Example of Type 1 Diabetes Mellitus

Diabetes mellitus type 1 (DM1) is notoriously a condition whose management in adult patients is totally autonomous, while for the pediatric age, it is reserved for the parents of young patients, with autonomy acquired progressively in adolescence. The management of DM1, in fact, involves, on the one hand, subcutaneous insulin therapy (by means of multi-day injections or insulin pumps) and, on the other hand, the continuous monitoring of blood glucose [37]. Although developments in recent years have greatly eased the burden of DM1 on parents and patients, acute adverse events (hyperglycemia and, especially, hypoglycemic episodes) remain relatively common in daily life. Knowing how to manage these events is crucial for any adult “caring” for a child with DM1, especially for younger age groups.

In this context, Gaslini’s Pediatric Diabetes Center has over twenty years of experience in training staff in schools where there are children with DM1. The meetings (at least 35–40 per year) take place in the schools, in the presence of a doctor and a nurse from the Diabetes Center, and deal with the most practical topics for the management of the child: the onset symptoms, the management of hyperglycemic episodes, the management of hypoglycemic episodes, the management of severe hypoglycemia, with the drafting of an emergency protocol, and lastly, the management of meals. In these meetings, there is always a practical demonstration on the use of Glucagon (life-saving drug in severe hypoglycemia).

The pandemic, with the consequent stringent rules, made these meetings difficult to manage in attendance. It was, therefore, decided to switch to web-based staff training. The following is the organization of the distance meetings:

- the school leader formally requests a meeting with the Pediatric Diabetes staff;
- the date and time of the meeting are decided (normally at the end of school activities);
- the manager shares the link for the video call with the health staff and teachers of the student with DM1;
- on the day of the meeting, the issues described above are discussed;
- the practical demonstration, which might have been somewhat perplexing via the web, has been made even easier by the recent release of intranasal glucagon, limiting the intramuscular formulation to children under age 4;
- normally the meeting is recorded by the school and made available even to those not present.

We can conclude that educational telemedicine allows for the continuation of the training of school personnel, which is essential to ensure that children with DM1 have a peaceful schooling. This modality, although it does not replace in-person meetings in their entirety, can continue particularly for those educational institutions that are difficult to reach or for more urgent meetings. This modality can be extended to other settings, such as sports clubs or scout groups for example. The technology applied to DM1 management has certainly facilitated these web meetings, which only a few years ago would have been difficult to sustain.

## 3. Emergencies

### 3.1. Remote Triage and Operation Centres

Pediatrics over the telephone is an established outpatient activity, especially in English-speaking countries, where it is codified and forms part of pediatrician and nurse training [38,39,40,41]. In the last two years, given the need to reduce in-person visits and hospital admissions, in order to limit the spread of contagions during a pandemic, there has been a considerable increase in the number of services provided by telemedicine, with the possibility of remotely managing non-serious cases at home. To date, the choice of management (telemedicine vs. outpatient vs. hospital) and the definition of urgency in the healthcare service have been made in an uncoded manner; indeed, indications for admission and red flags to be investigated by telephone often differ in the various centers. In this context, the priority areas of application, the organizational model, the information and training aspects, the performance indicators and the critical issues must be considered.

Remote telephone triage represents an indispensable tool for defining both the need for direct clinical assessment and the urgency and setting of care; this makes it possible, through the use of specific modalities (questions aimed at identifying red flags), to reduce the number of unnecessary visits and to optimize available local resources, both in terms of time and manpower [41]. The production of pediatric guidelines for carrying out telephone triage, with an indication of the specific elements to be investigated by telephone and by which modalities, is urgent in order to standardize healthcare services and access to hospital care throughout the country.

The organizational model must provide for families and pediatricians in the area so that they can quickly access the operational centers, where qualified personnel must effectively manage (remotely) the first and most delicate phase of emergency intervention [42,43].

Moreover, only qualified personnel specialized in the management of pediatric problems can perform effective telephone triage, identify the alarm bells, specific to pediatric age, and indicate the need for a prompt clinical assessment [41]. Therefore, the implementation of professional figures with specific training (pediatric nurses) in operational centers is urgently needed.

A far as performance indicators are concerned, a reduction in the improper use of emergency rooms and the number of unnecessary home care services performed by medical ambulances are the main objectives of the adequate implementation of telemedicine (telephone triage) in the operational centers [42]. Currently, remote triage is, in many realities, performed by staff not adequately trained in the management of children; adequate training must be provided on these issues for already tenured staff.

Telemedicine has important applications with regard to emergency-urgency management; this is the case both on the ground and in the context of non-pediatric emergency and admissions departments (DEAs), intervening in the stabilization of critically ill children [44].

In emergency/urgency, triage takes place through strict criteria and is managed by the 118 operations centers [45,46]. The staff of the 118 territorial emergency systems are involved on a daily basis in the management of pediatric patients, even though they do not have specific pediatric skills. The 118 emergency service in the territory intervenes in the pediatric patient and manages his or her transport to the nearest Department of Emergency (in the event of the impairment of the vital functions), or in their primary centralization to the pediatric Department of Emergency level II. If the child has direct self-preservation access or is initially stabilized at a non-specialist Department of Emergency, the problem of secondary centralization arises. Finally, children with special needs (tracheotomized, supported with mechanical ventilation at home, etc.) may require 118 intervention in the event of acute problems [47]. In this context, the possible applications of telemedicine are as follows:

- Remote assistance to rescue teams, including through augmented reality tools (e.g., glasses allowing the same view as the rescuers, and voice and sound transmission from the scene) [48,49,50,51];
- Triage support at the operations center to enable the most correct intervention [52,53];
- Support for non-specialist Department of Emergencies in setting up the diagnostic-therapeutic programme, favoring the timely centralization of serious cases with high developmental potential, but limiting the avoidable transfer of patients who can be effectively treated locally [54,55]
- Clinical follow-up of patients, either at home, at the Department of Emergency or at non-specialist pediatric facilities [56,57,58];
- In the event of a large influx of pediatric patients, it may happen that non-specialist personnel may have to intervene in the children’s case. Telemedicine can allow the remote specialist coordination of those dealing with the emergency. The same applies to highly diffusive infectious diseases [59,60,61,62,63,64].

This implies timely and effective responses. Hence, there is a need for 24/7 telemedicine control rooms.

### 3.2. Televisits, Teleconsultations and Telemonitoring

Despite the distancing and isolation imposed by the pandemic itself, during the COVID-19 era, the need to maintain a proximity in medicine, aimed at pediatric patients manifesting acute symptoms, prompted research and the development of alternative and integrated methodologies for an initial and in-depth assessment of the symptoms presented by the child [7]. This methodological approach was already the subject of studies and reflections in the pre-COVID era in situations where the pediatrician–patient–caregiver or pediatrician–consultant–patient proximity was hindered by particular environmental and/or clinical situations [43]. In these conditions, alternative and integrated strategies for an initial and in-depth assessment of the clinical condition presented by the patient can be identified in three types of intervention: (1) pediatrician/patient caregiver teleconsultation; (2) televisit and telemonitoring; (3) pediatrician/consultant specialist teleconsultation.

The pediatrician/caregiver teleconsultation can be developed by implementing, in an in-depth and structured way, the principles, methodologies, and techniques of telephone triage, consistent with the elements of good clinical practice from the most accredited scientific evidence. This practice has been in use for years among primary care pediatricians and is “facilitated” by the in-depth knowledge that the primary care pediatrician has of the child’s health and of the socio-cultural situation of his/her caregiver [62]. With the aim of extending the use of this practice to all possible actors (e.g., hospital pediatricians, the continuity of care physicians, consultant specialists), it is necessary to implement the health record for an adequate and structured sharing of the child’s and his/her caregiver’s socio-health situation.

Tele-consultation and telemonitoring can be adequately developed through the valorization and enhancement of communication systems, now widely diffused, that allow video-calling or the rapid sharing of image, audio, and audio–video files [62]. With such systems, parents, adequately guided by their primary care pediatrician or the other actors involved, can integrate the data collected through classic telephone counselling with the other important clinical elements necessary for the best definition of the presented case. Moreover, the dissemination and implementation of technological systems already on the market, capable of collecting and transmitting fundamental data for a better remote assessment (arterial oxygen saturation [SaO_2_], heart and respiratory rate, lung murmur, vision of the oral cavity, tympanic membranes, etc.) are essential.

Occasionally, the teleconsultation of the 2nd–3rd level specialist cannot be carried out at a time and place appropriate to the patient’s need, due to the logistical, environmental, or clinical situations of the patient. In this case, the teleconsultation, integrated with the anamnestic data, the results of the examinations and the clinical examination performed by the primary care pediatrician or hospital pediatrician, makes the response to the “health need” acutely expressed by the patient more effective and efficient.

### 3.3. Domestic Accidents

According to surveys from the Italian Institute for Statistics (ISTAT), more than 3 million domestic accidents occur every year in Italy [63], a figure that alone is enough to explain what a serious public health problem they represent. During the pandemic, in the pediatric age (already a favorite target of these), the number of emergencies increased with the time spent by children at home; in parallel, there was in an increase in the number of injuries and head injuries occurring within the home, especially those suspected to be caused by abuse/violence [64].

The use of telemedicine in this area can be useful through communication campaigns, educational interventions, parenting skills development programs, school programs, and surveillance and monitoring systems, consistent with the multi-level strategy of the Italian National Prevention Plan 2020–2025, in order to limit injuries in the home [65]. It is crucial to involve, in a transversal way, all the health services of interest to counter this phenomenon in the community, through integrated prevention actions and evidence-based interventions; the aims of these services are as follows:

- Ensure knowledge of the phenomenon and support for information flows based on the data collected;
- Monitor the population’s perception of risk and the frequency of domestic accidents, using current information flows and surveillance activities synergistically;
- Raise awareness of the risks of domestic accidents;
- Promote safety, with a focus on new patients and groups at greater risk: children, women and the elderly;
- Promote correct lifestyles with a focus on physical activity and the correct use of medication.

Even though technology can already enable us to provide effective telemedicine (thanks, for example, to advanced devices that allow the center operator to see the same things that a rescuer sees), for the time being, interest in prevention, rather than intervention, telemedicine prevails. At a later stage, however, after the population has been instructed on how to prevent problems, it must also be coached, in order to also explain it how to deal with these problems, should the need arise. Courses, such as Pediatric Basic Life Support–early Defibrillation (PBLSD) and airway defibrillation maneuvers, along with many other topics, could be the subject of an educational intervention that, by placing the citizen at the center, contributes to the realization of that empowerment about which so far much has been said, but little has been seen.

## 4. Discussion

Telemedicine is not a separate medical specialty, but is a tool that can be used to extend traditional practice beyond the usual physical spaces [66]. It is configured, in the general regulatory framework, as a different method of providing health and social-health services; therefore, it falls within the reference framework that regulates these processes with some clarifications on the conditions of implementation. A rigorous evaluation of telehealth services should include the use of health technology assessment (HTA) methods [67]. In this regard, the inclusion of indicators capable of describing performance, by considering the following aspects, is fundamental: size (volume of services provided), temporal continuity (duration and stability of the service), complexity (organizational complexity of the service), quality (standard and response performance of the service), efficiency (cost of the service), effectiveness (comparison with the population of users affected by the pathology covered by the telemedicine service but followed in a conventional manner, in the area of interest), and approval by patients and caregivers.

Taking into account the priorities of the health system and in line with the initiatives undertaken at an international level, a future priority is to create the enabling conditions for the dissemination of telemedicine services that are concretely integrated into clinical practice; with these effective responses to the changed health needs of citizens will be provided. For the coherent planning and use of these systems, it is necessary to identify, in the different contexts, the areas of application of telemedicine and the services that can be provided, also considering the relationships between the actors (patients/caregivers, doctors and other operators sanitary). To achieve this objective, information and training aspects, authorization and accreditation criteria, and ethical and regulatory aspects cannot be ignored. Unfortunately, limitations still include the absence of integrated, interoperable and fluid services with no barriers between the different care settings, as well as an appropriate use of information and training for all the players involved (i.e., patients, clinicians, technicians, IT specialists, managers).

## 5. Conclusions

The changes relating to the digital transformation underway in all sectors are unstoppable. For the digital transformation to take place in a productive sense, the contribution of not only all health professionals, but also of the patients is necessary. From this perspective, authors from different backgrounds were involved in the drafting of this Consensus, and, in the future, it is expected that other figures will be involved, primarily patients. In fact, the vision of connected care sees that the citizen/patient is engaged in actively participating in the treatment path so that they are assisted in a personalized, predictive and preventive way. The future scenario must be able to provide for the involvement of patients from the initial stages of planning any treatment path, even in the pediatric age, and increasing, where possible, the proximity of the health service to families.

## Figures and Tables

**Table 1:** Indicators for in-person examination with assessment of vital parameters.

Indications
All patients aged <3 months with
• Fever > 38 °C • Diarrhea and/or vomiting and/or poor nutrition to assess possible need for intravenous rehydration • Pallor reported by parents
All patients regardless of their age
• Moderate respiratory distress (breathlessness at rest, speaks in a broken manner, with few sentences, cannot lie supine, prefers to sit, presents moderate costo-sternal retractions, whistling is audible while breathing) • Cough > 7 days • Non compensating underlying disease (diabetes, metabolic diseases, adrenal insufficiency, renal insufficiency, liver failure, cystic fibrosis, ongoing immunosuppressive therapy, immunodeficiency) with fever and/or associated symptoms (diarrhea, vomiting, asthenia, rhinorrhea, cough, pharyngodynia, headache)

**Table 2:** Indications for hospitalization.

Indications for Admission
**Absolute** Fever in patients aged ≤3 months Persistence of high-grade fever (>38.5 °C) over 5 days O_2_ saturation <92% or signs of respiratory distress or tachypnoea: • 0–2 months = 60 acts/min • 2–12 months = 50 acts/min • 1–5 years = 40 acts/min • >5 years = 20 acts/min Convulsions or neurological symptoms Lethargy, altered consciousness Need for parenteral treatment and procedures (e. g. antibiotic therapy, chemotherapy, transfusions, lumbar puncture) Surgical necessity and/or acute pain (e.g., renal colic, head trauma) Cyanogenic heart diseases Alteration of myocardial enzymes, coagulation, liver cytolysis indices or alteration of lactic dehydrogenase
**Related**
Age 3–12 months or chronic illness or obesity and at least one of the following: • Persistence of fever for 3–5 days • O_2_ saturation <94%-mild respiratory distress • Extrapulmonary complications • Co-infections • Prematurity (<34 weeks EG)–small for gestational age (<2000 g) • Relapse of chronic illness requiring hospital procedures (e.g., acidosis)

**Table 3:** Priorities on pediatric telerehabilitation projects.

Urgent	Deferrable
Consolidate telecounseling	Exploring digital therapies based on AI, VR/exergames and wearables
Integrating traditional protocols with telerehabilitation	Test traditional treatment substitution on specific populations
Identify specific digital rehabilitation protocols	
Certification and definition or selection criteria for telerehabilitation and telemonitoring devices	
Deepening evidence and techniques for patient and caregiver engagement	

AI, artificial intelligence; VR, virtual reality.

## Data Availability

Not applicable.

## References

[1] World Health Organization Life Expectancy and Healthy Life Expectancy. https://www.who.int/data/gho/data/themes/mortality-and-global-health-estimates/ghe-life-expectancy-and-healthy-life-expectancy.

[2] Neves A.L., Burgers J. (2022). Digital technologies in primary care: Implications for patient care and future research. Eur. J. Gen. Pract..

[3] American Medical Association AMA Telehealth Quick Guide. https://www.ama-assn.org/practice-management/digital/ama-telehealth-quick-guide.

[4] Krupinski E.A., Bernard J. (2014). Standards and Guidelines in Telemedicine and Telehealth. Healthcare.

[5] Pappalardo M., Fanelli U., Chiné V., Neglia C., Gramegna A., Argentiero A., Esposito S. (2021). Telemedicine in Pediatric Infectious Diseases. Children.

[6] Vicente M.A., Fernández C., Guilabert M., Carrillo I., Martín-Delgado J., Mira J.J., Prometeo Working Group (2022). Patient Engagement Using Telemedicine in Primary Care during COVID-19 Pandemic: A Trial Study. Int. J. Environ. Res. Public Health.

[7] Istituto Superiore di Sanità Rapporto COVID Indicazioni ad Interim per Servizi Sanitari di Telemedicina in Pediatria Durante e Oltre la Pandemia COVID 19. https://www.iss.it/documents/20126/0/Rapporto+ISS+COVID-19+60_2020.pdf/6b4dfc13-fc37-fadd-3388-b93aef43a15d?t=1602857089054.

[8] Ministero Della Sanità (2022). Linee Guida Organizzative Contenenti il Modello Digitale per l’Attuazione dell’Assistenza Domiciliare. Milestone EU M6C1-4. https://www.salute.gov.it/imgs/C_17_pagineAree_5874_0_file.pdf.

[9] Matiz L.A., Kostacos C., Robbins-Milne L., Chang S.J., Rausch J.C., Tariq A. (2021). Integrating Nurse Care Managers in the Medical Home of Children with Special Health Care needs to Improve their Care Coordination and Impact Health Care Utilization. J. Pediatr. Nurs..

[10] Smaradottir B.F., Berntsen G.K.R., Fensli R.W. (2020). How to Enhance Digital Support for Cross-Organisational Health Care Teams? A User-Based Explorative Study. J. Health Eng..

[11] Bala N., Price S.N., Horan C.M., Gerber M.W., Taveras E.M. (2019). Use of Telehealth to Enhance Care in a Family-Centered Childhood Obesity Intervention. Clin. Pediatr..

[12] Provenzi L., Grumi S., Borgatti R. (2020). Alone with the Kids: Tele-Medicine for Children With Special Healthcare Needs During COVID-19 Emergency. Front. Psychol..

[13] Capello F., Pili A.E., Naimoli G. (2014). Telemedicine for Children’s Health.

[14] Naimoli G., Capello F. (2022). La cura delle informazioni digitali in telepediatria alla luce della normativa europea. Quaderni. ACP.

[15] Tozzi A.E., Carloni E., Gesualdo F., Russo L., Raponi M. (2015). Attitude of families of patients with genetic diseases to use m-health technologies. Telemed. J. E Health.

[16] Myers K., Vander Stoep A., Zhou C., McCarty C.A., Katon W. (2015). Effectiveness of a telehealth service delivery model for treating attention-deficit/hyperactivity disorder: A community-based randomized controlled trial. J. Am. Acad. Child. Adolesc. Psychiatry.

[17] Vander Stoep A., McCarty C.A., Zhou C., Rockhill C.M., Schoenfelder E.N., Myers K. (2017). The Children’s Attention-Deficit Hyperactivity Disorder Telemental Health Treatment Study: Caregiver Outcomes. J. Abnorm. Child. Psychol..

[18] Lustig T.A. (2012). The Role of Telehealth in an Evolving Health Care Environment: Workshop Summary.

[19] (2021). Indicazioni Nazionali per l’erogazione di Prestazioni e Servizi di Teleriabilitazione da Parte Delle Professioni Sanitarie. Conferenza Stato Regioni. https://www.statoregioni.it/media/4271/p-1-csr-atto-rep-n-231-18nov2021.pdf.

[20] Kim C.T., Greenberg J., Kim H. (2013). Pediatric rehabilitation: Trends in length of stay. J. Pediatr. Rehabil. Med..

[21] Rabatin A.E., Lynch M.E., Severson M.C., Brandenburg J.E., Driscoll S.W. (2020). Pediatric telerehabilitation medicine: Making your virtual visits efficient, effective and fun. J. Pediatr. Rehabil. Med..

[22] Tenforde A.S., Borgstrom H., Polich G., Steere H., Davis I.S., Cotton K., O’Donnell M., Silver J.K. (2020). Outpatient Physical, Occupational, and Speech Therapy Synchronous Telemedicine: A Survey Study of Patient Satisfaction with Virtual Visits During the COVID-19 Pandemic. Am. J. Phys. Med. Rehabil..

[23] Alonazi A. (2021). Effectiveness and Acceptability of Telerehabilitation in Physical Therapy during COVID-19 in Children: Findings of a Systematic Review. Children.

[24] Mani S., Sharma S., Omar B., Paungmali A., Joseph L. (2017). Validity and reliability of Internet-based physiotherapy assessment for musculoskeletal disorders: A systematic review. J. Telemed. Telecare..

[25] Grona S.L., Bath B., Busch A., Rotter T., Trask C., Harrison E. (2018). Use of videoconferencing for physical therapy in people with musculoskeletal conditions: A systematic review. J. Telemed. Telecare..

[26] Camden C., Pratte G., Fallon F., Couture M., Berbari J., Tousignant M. (2020). Diversity of practices in telerehabilitation for children with disabilities and effective intervention characteristics: Results from a systematic review. Disabil. Rehabil..

[27] Corti C., Oldrati V., Oprandi M.C., Ferrari E., Poggi G., Borgatti R., Urgesi C., Bardoni A. (2019). Remote Technology-Based Training Programs for Children with Acquired Brain Injury: A Systematic Review and a Meta-Analytic Exploration. Behav. Neurol..

[28] Zhang Y., Li R., Miao X., Cheng L.J., Lau Y. (2022). Virtual motor training to improve the activities of daily living, hand grip, and gross motor function among children with cerebral palsy: Meta-regression analysis. Gait Posture.

[29] Pietruszewski L., Burkhardt S., Yoder P.J., Heathcock J., Lewandowski D.J., Maitre N.L. (2020). Protocol and Feasibility-Randomized Trial of Telehealth Delivery for a Multicomponent Upper Extremity Intervention in Infants with Asymmetric Cerebral Palsy. Child. Neurol. Open..

[30] Wang T.N., Chen Y.L., Shieh J.Y., Chen H.L. (2021). Commercial Exergaming in Home-Based Pediatric Constraint-Induced Therapy: A Randomized Trial. OTJR.

[31] Medina-Mirapeix F., Lillo-Navarro C., Montilla-Herrador J., Gacto-Sanchez M., Franco-Sierra M.A., Escolar-Reina P. (2017). Predictors of parents’ adherence to home exercise programs for children with developmental disabilities, regarding both exercise frequency and duration: A survey design. Eur. J. Phys. Rehabil. Med..

[32] (2020). Report of the WCPT/INPTRA Digital Physical Therapy Practice Task Force. https://world.physio/sites/default/files/2020-06/WCPT-INPTRA-Digital-Physical-Therapy-Practice-Task-force-March2020.pdf.

[33] Surana B.K., Ferre C.L., Dew A.P., Brandao M., Gordon A.M., Moreau N.G. (2019). Effectiveness of Lower-Extremity Functional Training (LIFT) in Young Children with Unilateral Spastic Cerebral Palsy: A Randomized Controlled Trial. Neurorehabil. Neural. Repair..

[34] Kaelin V.C., Valizadeh M., Salgado Z., Parde N., Khetani M.A. (2021). Artificial Intelligence in Rehabilitation Targeting the Participation of Children and Youth with Disabilities: Scoping Review. J. Med. Internet. Res..

[35] Vibhuti H., Kumar N., Kataria C. (2021). Efficacy assessment of virtual reality therapy for neuromotor rehabilitation in home environment: A systematic review. Disabil. Rehabil. Assist. Technol..

[36] Lau R., Cheuk K.Y., Tam E., Hui S., Cheng J., Lam T.P. (2021). Feasibility and effects of 6-month home-based digitally supported E-Fit program utilizing high-intensity interval exercises in girls with adolescent idiopathic scoliosis: A randomized controlled pilot study. Stud. Health Technol. Inform..

[37] Blonde L., Umpierrez G.E., Reddy S.S., McGill J.B., Berga S.L., Bush M., Chandrasekaran S., DeFronzo R.A., Einhorn D., Galindo R.J. (2022). American Association of Clinical Endocrinology Clinical Practice Guideline: Developing a Diabetes Mellitus Comprehensive Care Plan-2022 Update. Endocr. Pract..

[38] Institute of Medicine (1996). Telemedicine: A Guide to Assessing Telecommunication in Health Care.

[39] World Health Organization (1997). A health telematics policy in support of WHO’s health-for-all strategy for global health development. Report of the International Consultation.

[40] American Telemedicine Association What Is Telemedicine?. https://www.americantelemed.org/ata-news/what-is-telemedicine-exactly/.

[41] Sood S., Mbarika V., Jugoo S., Dookhy R., Doarn C.R., Prakash N., Merrell R.C. (2007). What is telemedicine? A collection of 104 peer-reviewed perspectives and theoretical underpinnings. Telemed. J. E Health.

[42] Kane-Gill S.L., Rincon F. (2019). Expansion of Telemedicine Services: Telepharmacy, Telestroke, Teledialysis, Tele-Emergency Medicine. Crit. Care Clin..

[43] Burke B.L., Hall R.W. (2015). Section on telehealth care. Telemedicine: Pediatric Applications. Pediatrics.

[44] Sheikhtaheri A., Kermani F. (2018). Telemedicine in Diagnosis, Treatment and Management of Diseases in Children. Stud. Health. Technol. Inform..

[45] Ministero Della Salute Piano Per il Miglioramento del Sistema di Emergenza/Urgenza. Piano-Traccia Generale. https://www.salute.gov.it/imgs/C_17_pubblicazioni_856_allegato.pdf.

[46] Ministero Della Salute Piano per il Miglioramento del Sistema di Emergenza/Urgenza. Piano-Rete Ospedaliera. https://www.salute.gov.it/imgs/C_17_pubblicazioni_856_ulterioriallegati_ulterioreallegato_0_alleg.pdf.

[47] Ministero Della Salute Piano per il Miglioramento del Sistema di Emergenza/Urgenza. Piano-Urgenza Pediatrica. https://www.salute.gov.it/imgs/C_17_pubblicazioni_856_ulterioriallegati_ulterioreallegato_1_alleg.pdf.

[48] Munzer B.W., Khan M.M., Shipman B., Mahajan P. (2019). Augmented Reality in Emergency Medicine: A Scoping Review. J. Med. Internet. Res..

[49] Cicero M.X., Walsh B., Solad Y., Whitfill T., Paesano G., Kim K., Baum C.R., Cone D.C. (2015). Do you see what I see? Insights from using google glass for disaster telemedicine triage. Prehosp. Disaster. Med..

[50] Wang S., Parsons M., Stone-McLean J., Rogers P., Boyd S., Hoover K., Meruvia-Pastor O., Gong M., Smith A. (2017). Augmented Reality as a Telemedicine Platform for Remote Procedural Training. Sensors.

[51] Bifulco P., Narducci F., Vertucci R., Ambruosi P., Cesarelli M., Romano M. (2014). Telemedicine supported by Augmented Reality: An interactive guide for untrained people in performing an ECG test. Biomed. Eng. Online.

[52] Harvey J.B., Yeager B.E., Cramer C., Wheeler D., McSwain S.D. (2017). The Impact of Telemedicine on Pediatric Critical Care Triage. Pediatr. Crit. Care Med..

[53] Yang N.H., Dharmar M., Kuppermann N., Romano P.S., Nesbitt T.S., Hojman N.M., Marcin J.P. (2015). Appropriateness of disposition following telemedicine consultations in rural emergency departments. Pediatr. Crit. Care Med..

[54] Dharmar M., Romano P.S., Kuppermann N., Nesbitt T.S., Cole S.L., Andrada E.R., Vance C., Harvey D.J., Marcin J.P. (2013). Impact of critical care telemedicine consultations on children in rural emergency departments. Crit. Care Med..

[55] Heath B., Salerno R., Hopkins A., Hertzig J., Caputo M. (2009). Pediatric critical care telemedicine in rural underserved emergency departments. Pediatr. Crit. Care Med..

[56] Ferro F., Tozzi A.E., Erba I., Dall’Oglio I., Campana A., Cecchetti C., Geremia C., Rega M.L., Tontini G., Tiozzo E. (2021). Impact of telemedicine on health outcomes in children with medical complexity: An integrative review. Eur. J. Pediatr..

[57] Chuo J., Webster K.A. (2019). Practical use of telemedicine in the chronically ventilated infant. Semin. Fetal Neonatal. Med..

[58] Ambrosino N., Vitacca M., Dreher M., Isetta V., Montserrat J.M., Tonia T., Turchetti G., Winck J.C., Burgos F., Kampelmacher M. (2016). ERS Tele-Monitoring of Ventilator-Dependent Patients Task Force. Tele-monitoring of ventilator-dependent patients: A European Respiratory Society Statement. Eur. Respir. J..

[59] Litvak M., Miller K., Boyle T., Bedenbaugh R., Smith C., Meguerdichian D., Reisman D., Biddinger P., Licurse A., Goralnick E. (2022). Telemedicine Use in Disasters: A Scoping Review. Disaster. Med. Public Health Prep..

[60] Gilchrist N., Simpson J.N. (2019). Pediatric disaster preparedness: Identifying challenges and opportunities for emergency department planning. Curr. Opin. Pediatr..

[61] Burke R.V., Berg B.M., Vee P., Morton I., Nager A., Neches R., Wetzel R., Upperman J.S. (2012). Using robotic telecommunications to triage pediatric disaster victims. J. Pediatr. Surg..

[62] Doria M., Basile L., Cazzato T., Fiore M., Gulino A., Lamborghini A., Malorgio E., Ruggiero G., Spanevello V. (2020). Pediatria di Famiglia e Telemedicina. Il Medico. Pediatra.

[63] Istituto Nazionale di Statistica (2019). Aspetti della vita Quotidiana. Indagine Multiscopo. Roma: ISTAT. https://www.istat.it/it/archivio/91926.

[64] Sidpra J., Abomeli D., Hameed B., Baker J., Mankad K. (2021). Rise in the incidence of abusive head trauma during the COVID-19 pandemic. Arch. Dis. Child..

[65] Ministero Della Salute Piano Nazionale Della Prevenzione 2020–2025. https://www.salute.gov.it/imgs/C_17_notizie_5029_0_file.pdf.

[66] Smaradottir B.F., Fensli R.W. (2020). An Evaluation of Telemedicine Systems in Patient-Centred Care Teams. Stud. Health Technol. Inform..

[67] European Commission Health Technology Assessment—Overview. https://health.ec.europa.eu/health-technology-assessment/overview_en.

